# Crystal structure of ammonium/potassium *trans*-bis­(*N*-methyl­iminodi­acetato-κ^3^ *O*,*N*,*O*′)chromate(III) from synchrotron data

**DOI:** 10.1107/S2056989016011804

**Published:** 2016-07-22

**Authors:** Dohyun Moon, Jong-Ha Choi

**Affiliations:** a Pohang Accelerator Laboratory, POSTECH, Pohang 37673, Republic of Korea; b Department of Chemistry, Andong National University, Andong 36729, Republic of Korea

**Keywords:** crystal structure, synchrotron radiation, ammonium/potassium salt, bis­(methyl­iminodi­acetato)­chromate(III) ion, mida, *trans*-facial configuration, hydrogen bonding

## Abstract

The structure of the title compound, [(NH_4_)_0.8_K_0.2_][Cr(C_5_H_7_NO_4_)_2_] (C_5_H_7_NO_4_ is methyl­iminodi­acetate; mida), has been determined from synchrotron data. The Cr^III^ atom is located on a centre of symmetry and is coordinated by two N atoms and four O atoms of two facially arranged tridentate mida ligands, displaying a slightly distorted octa­hedral coordination environment. The Cr—N and mean Cr—O bond lengths are 2.0792 (14) and 1.958 (14) Å, respectively. The cation site is located on a twofold rotation axis and shows occupational disorder, being occupied by ammonium and potassium cations in a 0.8:0.2 ratio. In the crystal, inter­molecular hydrogen bonds involving the N—H groups of the ammonium cation as donor and the two non-coordinating O atoms of the carboxyl­ate group as acceptor groups consolidate the three-dimensional packing.

## Abstract 2

In the title complex, the Cr^III^ ion is coordinated to two methyl­iminodi­acetate (mida) dianions acting as tridentate ligands through the N atom and two O atoms of each carboxyl­ate group, in a distorted octa­hedral geometry. The partial ammonium cation is linked to two O atoms of carboxyl­ate group from neighboring mida groups through N—H⋯O hydrogen-bonding inter­actions.

## Chemical context   

Methyl­iminodi­acetate (abbreviated here as mida; C_5_H_7_NO_4_) can coordinate to a central metal ion as a tridentate ligand through one N atom and two O atoms. The mida ligand differs from iminodi­acetate (ida) in the substitution of the imino hydrogen with a methyl group. This change has significant consequences with respect to the configuration of the bis-chromate(III) complexes with these ligands. Two facial configurations in *cis* or *trans* mode relative to the two N atoms have been observed: for example K[Cr(ida)_2_]·3H_2_O (Mootz & Wunderlich, 1980 ▸) and Na[Cr(ida)_2_]·1.5H_2_O (Li *et al.*, 2003 ▸) are *cis-fac* structures whereas Na[Cr(mida)_2_] is a *trans-fac* structure (Suh *et al.*, 1997 ▸). However, the *trans* meridional isomer of octa­hedrally coordinated chromium(III) with ida or mida ligands has not yet been identified. In order to confirm the bonding mode of the methyl­iminodi­acetato ligand and its structural arrangement, we report herein on the crystal structure of the title salt, [(NH_4_)_0.8_K_0.2_][Cr(C_5_H_7_NO_4_)_2_], (I).
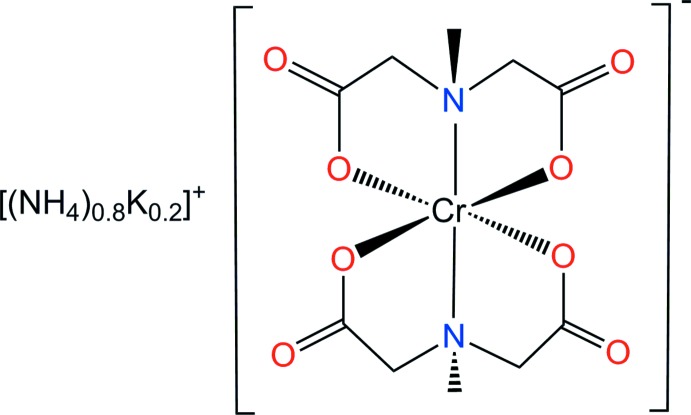


## Structural commentary   

**Figure 1:**
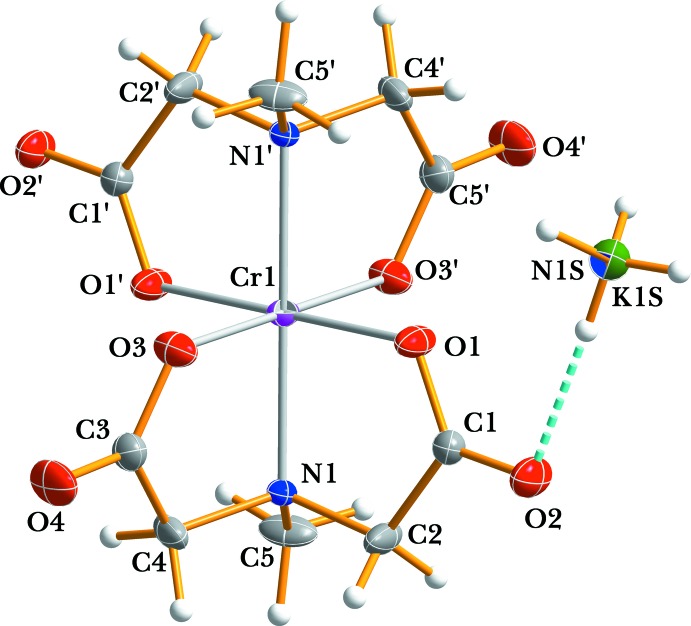
The structures of the mol­ecular entities of (I), showing the atom-numbering scheme. Non-H atoms are shown as displacement ellipsoids at the 50% probability level. The primed atoms are related by symmetry code (−*x* + 1, −*y* + 1, −*z* + 1). Dashed lines represent hydrogen-bonding inter­actions.

Counter-ionic species play important roles in crystal packings and hydrogen-bonding patterns. The structure reported here is another example of a [Cr(mida)_2_]^−^ salt but with a different cation (Suh *et al.*, 1996 ▸, 1997 ▸). The structural analysis shows that the two tridentate mida dianions octa­hedrally coordinate to the Cr^III^ metal atom through one N atom and two carboxyl­ate O atoms in a facial configuration. The coordinating N atoms are mutually *trans* due to point group 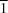 for the entire anionic complex. The asymmetric unit of (I) comprises one half of the Cr^III^ complex anion and one occupationally disordered ammonium/potassium cation (situated on a twofold rotation axis), respectively. An ellipsoid plot of title compound together with the atomic numbering is illustrated in Fig. 1 ▸.

The facial configuration of the complex anion in (I) can be compared with that of NH_4_[Cr(pydc)_2_] (pydc = pyridine-2,6-di­carboxyl­ate; Moon & Choi, 2015 ▸) where it displays a *trans* meridional configuration. The Cr—N and mean Cr—O bond lengths involving the mida ligands are 2.0792 (14) and 1.958 (14) Å, respectively, in good agreement with the values observed for Na[Cr(mida)_2_] (Suh *et al.*, 1997 ▸). Bond angles about the central chromium atom are 90.23 (6) for O1—Cr1—O3, 84.66 (6) for O1—Cr1—N1 and 82.62 (5)° for N1—Cr1—O3 indicating a distorted octa­hedral coordination environment. The C–O bond lengths within the carboxyl­ate group of the mida ligand range from 1.219 (2) to 1.296 (2) Å and can be compared with values of 1.225 (15) and 1.294 (15) Å for NH_4_[Cr(pydc)_2_] (Moon & Choi, 2015 ▸). The slightly longer C—O bond length (C1—O2 and C3—O4) and smaller O—C—O bond angles of the carboxyl­ate groups in the mida ligand of (I) compared to the ligand in Na[Cr(mida)_2_] (Suh *et al.*, 1997 ▸) may be attributed to the involvement of the two non-coordinating O atoms in hydrogen bonds with the N—H groups of the ammonium cation. The N—C and C—C distances in the mida moieties are close to those found in the free H_2_mida mol­ecule (Shkol’nikova *et al.*, 1986 ▸) and are equal to 1.479 (2)–1.494 (2) and 1.508 (3)–1.512 (2) Å, respectively.

## Supra­molecular features   

**Figure 2:**
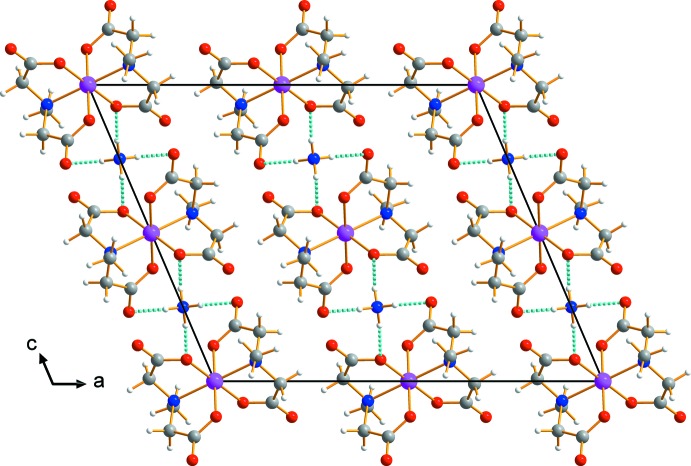
A packing diagram of (I), viewed perpendicular to the *ac* plane. Dashed lines represent hydrogen-bonding inter­actions N—H⋯O (cyan).

The pattern of hydrogen bonding around the cation is different from the crystal packing network in the related sodium salt (Suh *et al.*, 1996 ▸, 1997 ▸). The cation is linked to four non-coordinating O atoms of carboxyl­ate groups from four neighboring mida ligands through classical N—H⋯O hydrogen bonds (Table 1 ▸). An array of these inter­actions generate a three-dimensional network of mol­ecules whereby individual mol­ecules are stacked along the *b*-axis direction (Fig. 2 ▸).

## Database survey   

A search of the Cambridge Structural Database (Version 5.37, Feb. 2016 with two updates; Groom *et al.*, 2016 ▸) gave just two hits for a complex anion [Cr(C_5_H_7_NO_4_)_2_]^−^ unit. The crystal structures of Na[Cr(mida)_2_] with three different space groups have been reported and compared previously (Suh *et al.*, 1996 ▸, 1997 ▸).

## Synthesis and crystallization   

All chemicals were reagent-grade materials and were used without further purification. The starting material, K[Cr(mida)_2_] was prepared by a method similar to that outlined previously (Wernicke *et al.*, 1977 ▸; Uehara *et al.*, 1970 ▸). The potassium salt (0.25 g) was dissolved in 15 mL of water at 343 K and added to 5 mL of water containing 0.50 g of NH_4_Cl. The resulting solution was filtered to remove any impurities and allowed to stand at room temperature for several days to give pale pink plate-like crystals of the mixed-occupancy ammonium/potassium salt, (I), suitable for X-ray diffraction analysis.

## Refinement   

Crystal data, data collection and structure refinement details are summarized in Table 2 ▸. All H atoms of the complex were placed in geometrically idealized positions and constrained to ride on their parent atoms, with C—H distances of 0.97–0.98 Å and with *U*_iso_(H) values of 1.5 (meth­yl) and 1.2 times *U*_eq_ (all others) of the parent atoms. The H atoms of the cation were located from difference Fourier maps and refined with DFIX and DANG restraints and fixed N—H distances of 0.855 (9) and 0.869 (9) Å, with *U*_iso_(H) values of 1.2*U*_eq_(N). The occupancy of mixed-occupied (NH_4_/K) first was refined and then fixed at a ratio of 0.8:0.2. The corresponding (NH_4_/K) sites was refined using EXYZ/EADP commands for the two atom types.

## Supplementary Material

Crystal structure: contains datablock(s) I. DOI: 10.1107/S2056989016011804/wm5304sup1.cif

Structure factors: contains datablock(s) I. DOI: 10.1107/S2056989016011804/wm5304Isup2.hkl

CCDC reference: 1494843

Additional supporting information: crystallographic information; 3D view; checkCIF report

## Figures and Tables

**Table 1:** Hydrogen-bond geometry (Å, °)

*D*—H⋯*A*	*D*—H	H⋯*A*	*D*⋯*A*	*D*—H⋯*A*
N1*S*—H1*NS*⋯O3^i^	0.92 (1)	2.07 (1)	2.9658 (16)	166 (3)
N1*S*—H2*NS*⋯O2	0.90 (1)	1.95 (1)	2.8485 (17)	175 (3)

Symmetry code: (i) 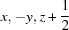.

**Table 2:** Experimental details

Crystal data	
Chemical formula	[(NH_4_)_0.8_K_0.2_][Cr(C_5_H_7_NO_4_)_2_]
*M*_r_	364.48
Crystal system, space group	Monoclinic, *C*2/*c*
Temperature (K)	243
*a*, *b*, *c* (Å)	16.786 (3), 6.5240 (13), 13.925 (3)
β (°)	113.19 (3)
*V* (Å^3^)	1401.8 (6)
*Z*	4
Radiation type	Synchrotron, λ = 0.610 Å
μ (mm^−1^)	0.61
Crystal size (mm)	0.02 × 0.02 × 0.01

Data collection	
Diffractometer	ADSC Q210 CCD area-detector
Absorption correction	Empirical (using intensity measurements) (*HKL3000sm *SCALEPACK**; Otwinowski & Minor, 1997 ▸)
*T*_min_, *T*_max_	0.989, 0.995
No. of measured, independent and observed [*I* > 2σ(*I*)] reflections	6984, 1833, 1519
*R*_int_	0.028
(sin θ/λ)_max_ (Å^−1^)	0.693

Refinement	
*R*[*F* ^2^ > 2σ(*F* ^2^)], *wR*(*F* ^2^), *S*	0.035, 0.100, 1.05
No. of reflections	1833
No. of parameters	110
No. of restraints	3
H-atom treatment	H atoms treated by a mixture of independent and constrained refinement
Δρ_max_, Δρ_min_ (e Å^−3^)	0.40, −0.69

Computer programs: *PAL BL2D-SMDC* (Shin *et al.*, 2016 ▸), *HKL3000sm* (Otwinowski & Minor, 1997 ▸), *SHELXT2014* (Sheldrick, 2015*a* ▸), *SHELXL2014* (Sheldrick, 2015*b* ▸), *DIAMOND* (Putz & Brandenburg, 2014 ▸) and *publCIF* (Westrip, 2010 ▸).

## supplementary crystallographic information

### Crystal data

**Table:**

[(NH_4_)_0.8_K_0.2_][Cr(C_5_H_7_NO_4_)_2_]	*F*(000) = 754
*M**_r_* = 364.48	*D*_x_ = 1.727 Mg m^−^^3^
Monoclinic, *C*2/*c*	Synchrotron radiation, λ = 0.610 Å
*a* = 16.786 (3) Å	Cell parameters from 23758 reflections
*b* = 6.5240 (13) Å	θ = 0.4–33.7°
*c* = 13.925 (3) Å	µ = 0.61 mm^−^^1^
β = 113.19 (3)°	*T* = 243 K
*V* = 1401.8 (6) Å^3^	Plate, pale pink
*Z* = 4	0.02 × 0.02 × 0.01 mm

### Data collection

**Table:**

ADSC Q210 CCD area-detector diffractometer	1519 reflections with *I* > 2σ(*I*)
Radiation source: PLSII 2D bending magnet	*R*_int_ = 0.028
ω scan	θ_max_ = 25.0°, θ_min_ = 2.7°
Absorption correction: empirical (using intensity measurements) (*HKL3000sm SCALEPACK*; Otwinowski & Minor, 1997)	*h* = −23→23
*T*_min_ = 0.989, *T*_max_ = 0.995	*k* = −9→9
6984 measured reflections	*l* = −17→17
1833 independent reflections	

### Refinement

**Table:**

Refinement on *F*^2^	Hydrogen site location: mixed
Least-squares matrix: full	H atoms treated by a mixture of independent and constrained refinement
*R*[*F*^2^ > 2σ(*F*^2^)] = 0.035	*w* = 1/[σ^2^(*F*_o_^2^) + (0.0702*P*)^2^] where *P* = (*F*_o_^2^ + 2*F*_c_^2^)/3
*wR*(*F*^2^) = 0.100	(Δ/σ)_max_ < 0.001
*S* = 1.05	Δρ_max_ = 0.40 e Å^−^^3^
1833 reflections	Δρ_min_ = −0.69 e Å^−^^3^
110 parameters	Extinction correction: SHELXL2014 (Sheldrick, 2015*b*), Fc^*^=kFc[1+0.001xFc^2^λ^3^/sin(2θ)]^-1/4^
3 restraints	Extinction coefficient: 0.0118 (19)

### Special details

**Table:**

Geometry. All esds (except the esd in the dihedral angle between two l.s. planes) are estimated using the full covariance matrix. The cell esds are taken into account individually in the estimation of esds in distances, angles and torsion angles; correlations between esds in cell parameters are only used when they are defined by crystal symmetry. An approximate (isotropic) treatment of cell esds is used for estimating esds involving l.s. planes.

### Fractional atomic coordinates and isotropic or equivalent isotropic displacement parameters (Å^2^)

**Table:**

	*x*	*y*	*z*	*U*_iso_*/*U*_eq_	Occ. (<1)
Cr1	0.5000	0.5000	0.5000	0.01108 (14)	
O1	0.53582 (8)	0.3160 (2)	0.61977 (10)	0.0228 (3)	
O2	0.64463 (9)	0.2272 (2)	0.76630 (10)	0.0267 (3)	
O3	0.54917 (8)	0.3115 (2)	0.42746 (10)	0.0228 (3)	
O4	0.65906 (10)	0.2681 (2)	0.37913 (13)	0.0361 (4)	
N1	0.62700 (8)	0.6057 (2)	0.56638 (10)	0.0129 (3)	
C1	0.61393 (11)	0.3333 (3)	0.68756 (13)	0.0175 (3)	
C2	0.67027 (13)	0.4929 (3)	0.66707 (15)	0.0326 (5)	
H2A	0.6886	0.5922	0.7245	0.039*	
H2B	0.7225	0.4260	0.6668	0.039*	
C3	0.62435 (11)	0.3600 (3)	0.42841 (13)	0.0196 (4)	
C4	0.66622 (12)	0.5484 (3)	0.49128 (15)	0.0232 (4)	
H4A	0.7283	0.5226	0.5297	0.028*	
H4B	0.6601	0.6633	0.4435	0.028*	
C5	0.63441 (13)	0.8296 (3)	0.58441 (19)	0.0333 (5)	
H5A	0.6948	0.8699	0.6086	0.050*	
H5B	0.6121	0.8653	0.6368	0.050*	
H5C	0.6012	0.9003	0.5197	0.050*	
K1S	0.5000	−0.0201 (2)	0.7500	0.0275 (3)	0.2
N1S	0.5000	−0.0201 (2)	0.7500	0.0275 (3)	0.8
H1NS	0.5139 (17)	−0.091 (4)	0.8115 (14)	0.033*	0.8
H2NS	0.5476 (13)	0.052 (4)	0.757 (2)	0.033*	0.8

### Atomic displacement parameters (Å^2^)

**Table:**

	*U*^11^	*U*^22^	*U*^33^	*U*^12^	*U*^13^	*U*^23^
Cr1	0.00847 (19)	0.01210 (19)	0.0120 (2)	−0.00089 (12)	0.00332 (13)	−0.00138 (12)
O1	0.0173 (6)	0.0248 (6)	0.0209 (6)	−0.0064 (5)	0.0018 (5)	0.0078 (5)
O2	0.0231 (7)	0.0300 (7)	0.0217 (7)	0.0003 (5)	0.0030 (5)	0.0107 (5)
O3	0.0201 (6)	0.0223 (6)	0.0290 (7)	−0.0052 (5)	0.0130 (5)	−0.0119 (5)
O4	0.0328 (8)	0.0430 (9)	0.0416 (9)	−0.0005 (7)	0.0243 (7)	−0.0173 (7)
N1	0.0110 (6)	0.0145 (6)	0.0134 (6)	−0.0023 (5)	0.0050 (5)	−0.0013 (5)
C1	0.0174 (8)	0.0198 (8)	0.0152 (8)	−0.0010 (6)	0.0063 (6)	0.0000 (6)
C2	0.0205 (9)	0.0530 (14)	0.0160 (9)	−0.0150 (8)	−0.0018 (7)	0.0125 (8)
C3	0.0201 (8)	0.0219 (8)	0.0185 (9)	0.0005 (6)	0.0094 (6)	−0.0027 (6)
C4	0.0203 (8)	0.0327 (9)	0.0226 (10)	−0.0081 (7)	0.0148 (7)	−0.0083 (7)
C5	0.0221 (9)	0.0186 (9)	0.0574 (14)	−0.0070 (7)	0.0137 (9)	−0.0129 (8)
K1S	0.0277 (7)	0.0278 (8)	0.0270 (8)	0.000	0.0107 (6)	0.000
N1S	0.0277 (7)	0.0278 (8)	0.0270 (8)	0.000	0.0107 (6)	0.000

### Geometric parameters (Å, º)

**Table:**

Cr1—O1	1.9479 (13)	C2—H2B	0.9800
Cr1—O1^i^	1.9479 (13)	C3—C4	1.512 (2)
Cr1—O3	1.9673 (12)	C3—K1S^ii^	3.371 (2)
Cr1—O3^i^	1.9673 (12)	C4—H4A	0.9800
Cr1—N1	2.0792 (14)	C4—H4B	0.9800
Cr1—N1^i^	2.0792 (14)	C5—H5A	0.9700
O1—C1	1.284 (2)	C5—H5B	0.9700
O1—K1S	3.0524 (17)	C5—H5C	0.9700
O2—C1	1.226 (2)	K1S—O2^iii^	2.8484 (16)
O2—K1S	2.8485 (17)	K1S—O3^iv^	2.9658 (16)
O3—C3	1.296 (2)	K1S—O3^ii^	2.9658 (16)
O3—K1S^ii^	2.9658 (16)	K1S—O4^iv^	3.033 (2)
O4—C3	1.219 (2)	K1S—O4^ii^	3.033 (2)
O4—K1S^ii^	3.033 (2)	K1S—O1^iii^	3.0524 (17)
N1—C5	1.479 (2)	K1S—C1^iii^	3.322 (2)
N1—C4	1.486 (2)	K1S—C3^ii^	3.371 (2)
N1—C2	1.494 (2)	K1S—C3^iv^	3.371 (2)
C1—C2	1.508 (3)	N1S—H1NS	0.920 (10)
C1—K1S	3.322 (2)	N1S—H2NS	0.899 (10)
C2—H2A	0.9800		
			
O1—Cr1—O1^i^	180.00 (5)	O2—K1S—O3^ii^	112.23 (4)
O1—Cr1—O3	90.23 (6)	O3^iv^—K1S—O3^ii^	100.26 (7)
O1^i^—Cr1—O3	89.77 (6)	O2^iii^—K1S—O4^iv^	150.81 (4)
O1—Cr1—O3^i^	89.77 (6)	O2—K1S—O4^iv^	74.40 (4)
O1^i^—Cr1—O3^i^	90.23 (6)	O3^iv^—K1S—O4^iv^	43.35 (4)
O3—Cr1—O3^i^	180.0	O3^ii^—K1S—O4^iv^	92.48 (6)
O1—Cr1—N1	84.66 (6)	O2^iii^—K1S—O4^ii^	74.40 (4)
O1^i^—Cr1—N1	95.34 (6)	O2—K1S—O4^ii^	150.82 (4)
O3—Cr1—N1	82.62 (5)	O3^iv^—K1S—O4^ii^	92.48 (6)
O3^i^—Cr1—N1	97.38 (5)	O3^ii^—K1S—O4^ii^	43.36 (4)
O1—Cr1—N1^i^	95.34 (6)	O4^iv^—K1S—O4^ii^	115.53 (8)
O1^i^—Cr1—N1^i^	84.66 (6)	O2^iii^—K1S—O1^iii^	43.90 (4)
O3—Cr1—N1^i^	97.38 (5)	O2—K1S—O1^iii^	84.65 (6)
O3^i^—Cr1—N1^i^	82.62 (5)	O3^iv^—K1S—O1^iii^	91.19 (4)
N1—Cr1—N1^i^	180.00 (7)	O3^ii^—K1S—O1^iii^	154.19 (4)
C1—O1—Cr1	117.20 (11)	O4^iv^—K1S—O1^iii^	111.36 (5)
C1—O1—K1S	90.53 (10)	O4^ii^—K1S—O1^iii^	113.71 (4)
Cr1—O1—K1S	152.23 (6)	O2^iii^—K1S—O1	84.65 (6)
C1—O2—K1S	101.75 (11)	O2—K1S—O1	43.90 (4)
C3—O3—Cr1	116.48 (11)	O3^iv^—K1S—O1	154.19 (4)
C3—O3—K1S^ii^	96.63 (10)	O3^ii^—K1S—O1	91.19 (4)
Cr1—O3—K1S^ii^	142.48 (6)	O4^iv^—K1S—O1	113.71 (4)
C3—O4—K1S^ii^	95.27 (12)	O4^ii^—K1S—O1	111.36 (5)
C5—N1—C4	109.69 (15)	O1^iii^—K1S—O1	88.15 (7)
C5—N1—C2	110.46 (15)	O2^iii^—K1S—C1^iii^	21.17 (4)
C4—N1—C2	110.50 (15)	O2—K1S—C1^iii^	98.43 (6)
C5—N1—Cr1	113.95 (11)	O3^iv^—K1S—C1^iii^	103.11 (4)
C4—N1—Cr1	105.34 (10)	O3^ii^—K1S—C1^iii^	131.52 (4)
C2—N1—Cr1	106.75 (10)	O4^iv^—K1S—C1^iii^	132.69 (5)
O2—C1—O1	123.79 (16)	O4^ii^—K1S—C1^iii^	93.56 (4)
O2—C1—C2	119.04 (16)	O1^iii^—K1S—C1^iii^	22.73 (4)
O1—C1—C2	117.17 (15)	O1—K1S—C1^iii^	85.72 (6)
O2—C1—K1S	57.08 (10)	O2^iii^—K1S—C1	98.43 (6)
O1—C1—K1S	66.74 (9)	O2—K1S—C1	21.17 (4)
C2—C1—K1S	175.95 (12)	O3^iv^—K1S—C1	131.52 (4)
N1—C2—C1	114.11 (15)	O3^ii^—K1S—C1	103.11 (4)
N1—C2—H2A	108.7	O4^iv^—K1S—C1	93.56 (4)
C1—C2—H2A	108.7	O4^ii^—K1S—C1	132.69 (5)
N1—C2—H2B	108.7	O1^iii^—K1S—C1	85.72 (6)
C1—C2—H2B	108.7	O1—K1S—C1	22.73 (4)
H2A—C2—H2B	107.6	C1^iii^—K1S—C1	92.12 (7)
O4—C3—O3	123.62 (17)	O2^iii^—K1S—C3^ii^	93.03 (5)
O4—C3—C4	120.65 (16)	O2—K1S—C3^ii^	133.81 (4)
O3—C3—C4	115.66 (15)	O3^iv^—K1S—C3^ii^	94.64 (6)
O4—C3—K1S^ii^	63.63 (11)	O3^ii^—K1S—C3^ii^	22.45 (4)
O3—C3—K1S^ii^	60.92 (9)	O4^iv^—K1S—C3^ii^	103.37 (7)
C4—C3—K1S^ii^	166.70 (13)	O4^ii^—K1S—C3^ii^	21.10 (4)
N1—C4—C3	112.21 (14)	O1^iii^—K1S—C3^ii^	134.49 (4)
N1—C4—H4A	109.2	O1—K1S—C3^ii^	104.15 (4)
C3—C4—H4A	109.2	C1^iii^—K1S—C3^ii^	113.34 (5)
N1—C4—H4B	109.2	C1—K1S—C3^ii^	121.12 (4)
C3—C4—H4B	109.2	O2^iii^—K1S—C3^iv^	133.81 (4)
H4A—C4—H4B	107.9	O2—K1S—C3^iv^	93.03 (5)
N1—C5—H5A	109.5	O3^iv^—K1S—C3^iv^	22.45 (4)
N1—C5—H5B	109.5	O3^ii^—K1S—C3^iv^	94.64 (6)
H5A—C5—H5B	109.5	O4^iv^—K1S—C3^iv^	21.10 (4)
N1—C5—H5C	109.5	O4^ii^—K1S—C3^iv^	103.37 (7)
H5A—C5—H5C	109.5	O1^iii^—K1S—C3^iv^	104.15 (4)
H5B—C5—H5C	109.5	O1—K1S—C3^iv^	134.49 (4)
O2^iii^—K1S—O2	111.01 (8)	C1^iii^—K1S—C3^iv^	121.12 (4)
O2^iii^—K1S—O3^iv^	112.23 (4)	C1—K1S—C3^iv^	113.34 (5)
O2—K1S—O3^iv^	110.35 (4)	C3^ii^—K1S—C3^iv^	97.73 (8)
O2^iii^—K1S—O3^ii^	110.35 (4)	H1NS—N1S—H2NS	105.9 (19)
			
K1S—O2—C1—O1	−1.9 (2)	K1S^ii^—O4—C3—C4	−165.65 (16)
K1S—O2—C1—C2	178.67 (16)	Cr1—O3—C3—O4	−173.25 (15)
Cr1—O1—C1—O2	−179.97 (14)	K1S^ii^—O3—C3—O4	−11.5 (2)
K1S—O1—C1—O2	1.73 (19)	Cr1—O3—C3—C4	3.7 (2)
Cr1—O1—C1—C2	−0.5 (2)	K1S^ii^—O3—C3—C4	165.51 (14)
K1S—O1—C1—C2	−178.83 (16)	Cr1—O3—C3—K1S^ii^	−161.79 (12)
Cr1—O1—C1—K1S	178.30 (12)	C5—N1—C4—C3	−151.02 (16)
C5—N1—C2—C1	127.82 (19)	C2—N1—C4—C3	86.96 (18)
C4—N1—C2—C1	−110.61 (19)	Cr1—N1—C4—C3	−27.99 (18)
Cr1—N1—C2—C1	3.4 (2)	O4—C3—C4—N1	−165.12 (17)
O2—C1—C2—N1	177.29 (16)	O3—C3—C4—N1	17.8 (2)
O1—C1—C2—N1	−2.2 (3)	K1S^ii^—C3—C4—N1	89.7 (5)
K1S^ii^—O4—C3—O3	11.2 (2)		

Symmetry codes: (i) −*x*+1, −*y*+1, −*z*+1; (ii) −*x*+1, −*y*, −*z*+1; (iii) −*x*+1, *y*, −*z*+3/2; (iv) *x*, −*y*, *z*+1/2.

### Hydrogen-bond geometry (Å, º)

**Table:**

*D*—H···*A*	*D*—H	H···*A*	*D*···*A*	*D*—H···*A*
N1*S*—H1*NS*···O3^iv^	0.92 (1)	2.07 (1)	2.9658 (16)	166 (3)
N1*S*—H2*NS*···O2	0.90 (1)	1.95 (1)	2.8485 (17)	175 (3)

Symmetry code: (iv) *x*, −*y*, *z*+1/2.
